# Implication of synaptotagmins 4 and 7 in activity-dependent somatodendritic dopamine release in the ventral midbrain

**DOI:** 10.1098/rsob.210339

**Published:** 2022-03-02

**Authors:** Benoît Delignat-Lavaud, Charles Ducrot, Willemieke Kouwenhoven, Nina Feller, Louis-Éric Trudeau

**Affiliations:** ^1^ Department of Pharmacology and Physiology, Université de Montréal, QC, Canada H3T 1J4; ^2^ Department of Neurosciences, Faculty of Medicine, Université de Montréal, QC, Canada H3T 1J4; ^3^ Neural Signaling and Circuitry Research Group (SNC), Montréal, QC, Canada H3C 3J7

**Keywords:** synaptotagmin, somatodendritic, dopamine, exocytosis, neurotransmission

## Abstract

Dopamine (DA) neurons can release DA not just from axon terminals, but also from their somatodendritic (STD) compartment through a mechanism that is still incompletely understood. Using voltammetry in mouse mesencephalic brain slices, we find that STD DA release has low capacity and shows a calcium sensitivity that is comparable to that of axonal release. We find that the molecular mechanism of STD DA release differs from axonal release with regard to the implication of synaptotagmin (Syt) calcium sensors. While individual constitutive knockout of Syt4 or Syt7 is not sufficient to reduce STD DA release, the removal of both isoforms reduces this release by approximately 50%, leaving axonal release unimpaired. Our work unveils clear differences in the mechanisms of STD and axonal DA release.

## Graphical abstract

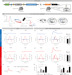

## 1. Introduction

Dopamine (DA) neurons of the mesencephalon play a key role in motor control, motivated behaviours and cognition [1,2]. DA neurons can release DA not only from axon terminals by a classical exocytotic mechanism [3,4], but also through their somatodendritic (STD) compartment, as demonstrated by multiple approaches including *in vivo* microdialysis, fast scan cyclic voltammetry (FSCV) and patch-clamp recordings of D2 receptor mediated currents in the ventral tegmental area (VTA) and substantia nigra pars compacta (SNc) [5–9]. Although there is limited direct evidence, STD DA release is believed to be implicated in regulating the excitability of DA neurons through activation of STD D2 autoreceptors [5]. It has also been suggested to regulate motor behaviours [10,11], mainly by local activation of D1 receptors.

The molecular mechanism of STD DA release is still unclear. Reversal of the DA transporter (DAT) has been proposed [12], but other studies support the involvement of a vesicular exocytotic-like mechanism, in agreement with the fact that STD DA release is activity-dependent [5,13,14], reserpine-sensitive [5,15,16], calcium-dependent [5,13,15,17–19], dependent on active zone proteins [20,21], quantal in nature [22,23] and blocked by botulinum neurotoxins, which disrupt SNARE proteins [13,24,25]. Although large pools of DA-containing small clear synaptic vesicles are not found in the dendrites of DA neurons, this cellular compartment contains pleiomorphic vesicles that bear the vesicular monoamine transporter (VMAT2), suggesting that they could be sites of DA storage in dendrites [26]. Together, these findings suggest that, although there may be some fundamental differences between the mechanisms of terminal and STD DA release, both implicate a form of exocytosis.

Although STD DA release is calcium-dependent, conflicting results exist regarding the calcium sensitivity of STD DA release in comparison with axonal release. Previous studies performed in guinea pig reported that STD DA release persists at extracellular calcium concentrations as low as 0.5 mM, a concentration at which axonal release is typically abrogated [13,17]. By contrast, previous work performed with mouse tissue and indirectly detecting STD DA release using the patch-clamp technique and STD D2 receptor activation, reported that axonal and STD DA release display a similar calcium dependency [5,19,23,27–29]. Here, we reexamined this question in mouse brain slices after optimizing direct detection of STD DA using FSCV.

Finally, an important outstanding question is the identification of the molecular mechanism of STD DA release. Building on previous *in vitro* work suggesting possible roles of synaptotagmin (Syt) 4 (Syt4) and Syt7 [3], in the present study we tested the hypothesis that Syt4 and Syt7 play a key role in STD DA release in the intact brain by quantifying STD DA release in Syt4, Syt7 and Syt4/7 double constitutive knockout (KO) mice.

## 2. Material and methods

### 2.1. Animals

Male and female mice of 11–12 weeks were used in this study. For optogenetic experiments, B6;129S-Gt(ROSA)26Sor^tm32(CAG-COP4*H134R/EYFP)Hze^/J (Ai32, The Jackson Laboratory, stock 012569, USA) homozygote mice expressing a floxed H134R variant of the light-activated channelrhodopsin-2 were bred with homozygote B6.SJL-Slc6a3^tm1.1(cre)Bkmn^/J (DAT^IREScre^, The Jackson Laboratory, stock 006660, USA) expressing the cre recombinase under control of the DAT promoter, allowing channelrhodospin-2 to be expressed selectively in DA neurons. Heterozygote DAT^IREScre^ mice were also used for experiments in which ChR2 was virally expressed. Constitutive KO mice for Syt4 (129S6.129X1(B6)-Syt4^tm1Hahe^/J, The Jackson Laboratory, stock #012400, USA) [30], Syt7 [31] and WT littermates were bred from heterozygous crosses or crossed with each other to obtain double KO mice. Genotyping for Syt4 KO mice was determined using specific primers to target the wild-type Syt4 sequence (primers Syt4WT-fwd and Syt4WT-rev) and the neomycin cassette within the mutated allele (primers neo-fwd and Syt4WT-rev)—Syt4WT-fwd: CACTTCCCTCACGTCAGAGGAG; Syt4WT-rev: GCAAGGAGAGCTCTTGGATGTG; neo-fwd: AACCACACTGCTCGACATTGGG. Genotyping for Syt7 KO mice was performed using specific primers to target the wild-type Syt7 sequence (Syt7WT-fwd: CATCCTCCACTGGCCATGAATG; Syt7WT-rev: GCTTCACCTTGGTCTCCAG) and the neomycin cassette within the mutated allele (neo-fwd: CTTGGGTGGAGAGGCTATTC; neo-rev: AGGTGAGATGACAGGAGATC), as provided by Jackson. Genotyping for Syt7 mutation in combined Syt4/7 KO mice was determined using another set of specific primers due to overlapping sequences within the neomycin cassette used in both the Syt4 and Syt7 mouse lines: neo-fwd: CTTGGGTGGAGAGGCTATTC and Syt7WTexon4: AGTGTCCAGGCTCCC. Experiments were performed blind with regard to animal genotype, with the exception of Syt4 KO mice, because these KO mice could be easily identified due to a neurodevelopmental alteration of the anterior commissure and corpus callosum (electronic supplementary material, figure S1C). Housing was at a constant temperature (21°C) and humidity (60%), under a fixed 12 h light/dark cycle, with food and water available ad libitum.

### 2.2. Stereotaxic injections

Six- to seven-week-old DAT^IREScre^ mice were anaesthetized with isoflurane (Aerrane; Baxter, Deerfield, IL, USA) and fixed on a stereotaxic frame (Stoelting,Wood Dale, IL, USA). A small hole was drilled in the exposed skull and a Hamilton syringe was used for the injections. For optogenetic experiments, an adeno-associated virus (AAV5-EF1a-DIO-hChR2(H134R)-EYFP, 4,2 × 10^12^ vg ml^−1^, UNC GTC Vector Core, USA) was injected bilaterally at the following injection coordinates [AP (anterior–posterior; ML (medial–lateral); DV (dorsal-ventral), from bregma], to infect neurons in the entire ventral mesencephalon: AP −3.0 mm; ML ± 1.0 mm; DV −4.5 mm. Animals recovered in their home cage and were closely monitored for 3 days. The animals were used one month after injection, allowing maximal expression of ChR2 in DA neurons. Success of the injection was visually validated each time during the slicing of the brains by visualizing the presence of the eYFP reporter.

### 2.3. Brain slice preparation and solutions

Acute brain slices from 11- to 12-week-old male or female mice were used for the FSCV recordings. When possible, matched pairs of WT and KO mice were used on each experimental day. The animals were anaesthetized with halothane, quickly decapitated and the brain harvested. Next, the brain was submersed in ice-cold oxygenated artificial cerebrospinal fluid (aCSF) containing (in mM): NaCl (125), KCl (2.5), KH_2_PO_4_ (0.3), NaHCO_3_ (26), glucose (10), CaCl_2_ (2.4), MgSO_4_ (1.3) and coronal VTA and/or striatal brain slices of 300 µm thickness were prepared with a VT1000S vibrating blade microtome. Once sliced, the tissue was transferred to oxygenated aCSF at room temperature and allowed to recover for at least 1 h. For recordings, slices were placed in a custom-made recording chamber superfused with aCSF at 1 ml min^−1^ and maintained at 32°C with a TC-324B single-channel heater controller (Warner Instruments, USA). All solutions were adjusted at pH 7.35–7.4, 300 mOsm kg^−1^ and saturated with 95% O_2_-5% CO_2_ at least 30 min prior to each experiment.

### 2.4. Fast scan cyclic voltammetry recordings

Optically or electrically evoked DA release was measured by FSCV using a 7 µm diameter carbon-fibre electrode placed into the tissue approximately 100 µm below the surface. A bipolar electrode (Plastics One, Roanoke, VA, USA) or an optical fibre connected to a 470 nm wavelength LED was placed approximately 200 µm away. Carbon-fibre electrodes were fabricated as previously described [32]. Briefly, carbon fibres (Goodfellow Cambridge Limited, UK) of 7 µm in diameter were aspirated into ethanol-cleaned glass capillaries (1.2 mm O.D., 0.68 mm I.D., 4 inches long; World Precision Instruments, FL, USA). The glass capillaries were then pulled using a P-2000 micropipette puller (Sutter Instruments, Novato, USA), dipped into 90°C epoxy for 30 s (Epo-Tek 301, Epoxy Technology, MASS, USA) and cleaned in hot acetone for 3 s. The electrodes were heated at 100°C for 12 h and 150°C for 5 days. Electrodes were polished and filed with potassium acetate at 4 M and potassium chloride at 150 mM. The protruding carbon fibres were cut using a scalpel blade under direct visualization to a length allowing to obtain maximal basal currents of 100 to 180 nA.

The electrodes were calibrated with 1 µM DA in aCSF before and after each recorded slice and the mean of the current values obtained were used to determine the amount of released DA. After use, electrodes were cleaned with isopropyl alcohol (Bioshop, Canada). The potential of the carbon-fibre electrode was scanned at a rate of 300 V s^−1^ according to a 10 ms triangular voltage wave (−400 to 1000 mV versus Ag/AgCl) with a 100 ms sampling interval, using a CV 203BU headstage preamplifier (Molecular Devices) and a Axopatch 200B amplifier (Molecular Devices, USA). Data were acquired using a Digidata 1440a analogue to digital converter board (Molecular Devices, USA) connected to a computer using Clampex (Molecular Devices, USA). Slices were left to stabilize for 20 min before any electrochemical recordings. After positioning of the bipolar stimulation electrode or the optical probe and carbon-fibre electrodes in the tissue, single pulses (400 µA or 30 mW, 1 ms) or pulse trains (30 pulses at 10 Hz) were applied to the tissue to trigger DA release. For evaluating the calcium dependency of axonal and STD release, variations of calcium concentrations in the aCSF (0, 0.5 and 2.4 mM) were compensated by changing the concentration of MgSO_4_ to keep divalent cation levels equivalent.

### 2.5. Immunohistochemistry

For Syt immunolabelling experiments, 40 µm brain slices from animals perfused with 4% paraformaldehyde (in PBS, pH-7.4) were cut with a cryostat (Leica CM 1800; Leica Canada) and used for immunohistochemistry (IHC). Because selective and specific Syt4, Syt7 and VMAT2 antibodies were all from the same host species (rabbit), a double labelling protocol adapted from Jackson ImmunoReseach (https://www.jacksonimmuno.com/technical/products/protocols/double-labeling-same-species-primary) with monovalent Fab fragments was used. After a PBS wash, the tissue was permeabilized, non-specific binding sites blocked (goat serum 5%) and incubated overnight at room temperature with the first primary antibody, rabbit anti-Syt1 (1/1000; #105002, Synaptic Systems, Germany), rabbit anti-Syt7 (1/1000; #105173, Synaptic Systems, Germany) or anti-Syt4 (1/1000; kind gift for Dr Mitsunori Fukuda, Japan), followed by 2 h with a first secondary antibody (rabbit Alexa Fluor-488–conjugated, 1 : 500, Invitrogen, Canada). A blocking step of antigenic sites from the first primary and secondary antibody combination was performed thereafter by a 3 h incubation with normal serum from the same species as the primary antibody, followed by a blocking solution (goat block: PBS, Triton X100 0.3%, bovine serum albumin 5%) with 50 µg ml^−1^ of unconjugated monovalent Fab fragments against the host of the primary antibody, overnight, at room temperature and under agitation. Slices were then washed, and a second labelling was performed with a second primary antibody (rabbit anti-VMAT2, 1 : 1000, gift of Dr Gary Miller, Columbia University), and a second secondary antibody (rabbit Alexa Fluor-546–conjugated, 1 : 500, Invitrogen). This VMAT2 antibody has been well-validated previously [33]. For each IHC staining, a control group was included, with the full protocol except for omission of the second primary antibody. A classical immunostaining protocol was used for the KO validation of Syt4 and Syt7 antibodies (electronic supplementary material, figure S1), using mouse anti-tyrosine hydroxylase (Millipore Sigma; 1 : 1000) and rabbit anti-Syt4 or anti-Syt7 primary antibodies (Synaptic Systems; 1 : 1000) subsequently detected using Alexa Fluor-488-conjugated and Alexa Fluor-546-conjugated secondary antibodies (Invitrogen; 1 : 500).

### 2.6. Confocal imaging

Images were acquired using an Olympus Fluoview FV1000 point-scanning confocal microscope (Olympus, Canada) with a 60 x oil-immersion objective (NA 1.35). Images acquired using 488 nm and 546 nm laser excitation were scanned sequentially to prevent non-specific bleed-through signal. All image analysis was performed using ImageJ (National Institutes of Health) software.

### 2.7. Reverse transcriptase-quantitative PCR

We used RT-qPCR to quantify the amount of mRNA encoding Syt1, 4, 5, 7 and 11 in brain tissue from P70 Syt4^+^/^+^ and Syt4^−^/^−^ mice and P70 Syt7^+^/^+^ and Syt7^−^/^−^ mice. Adult whole brains were harvested and homogenized in Trizol solution, then RNA extraction was performed using RNAeasy Mini Kit (Quiagen, Canada) according to the manufacturer's instructions. Total purified RNA (40 ng) was reverse-transcribed in a total of 20 µl including 1 µl of dNTP, 1 µl of random hexamer, 4 µl of 5 X buffer 5 X, 2 µl of dithiothreitol, 1 µl of RNAse-Out and 1 µl of the Moloney Murine Leukemia Virus reverse transcriptase enzyme (MML-V, Invitrogen). Quantitative PCR was carried out in a total of 15 µl consisting of 3 µl cDNA, 7.5 µl SYBER green PCR master mix (Quanta Biosciences, USA), 10 µM of each primer and completed up to 15 µl with RNA-free water. qPCR was performed on a Light Cycler 96 machine (Roche, Canada) using the following procedure: 10 min at 95°C; 40 cycles of 30 s at 95°C, 40 s at 57°C and 40 s at 72°C; 1 cycle of 15 s at 95°C, 15 s at 55°C and 15 s at 95°C. Results were analysed with Light Cycler 96 software and Excel. Primers used for qPCR were as follows: Syt1: 5′ GTGGCAAGACACTGGTGAT 3′ and 5′ CTCAGGACTCTGGAGATCG 3′; Syt4: 5′ CACTTCCCTCACGTCAGAGGAG 3′ and 5′ GCAAGGAGAGCTCTTGGATGTG 3′; Syt5: 5′ GTCCCATACGTGCAACTAGG 3′ and 5′ AACGGAGAGAGAAGCAGATG 3′; Syt7: 5′ CCAGACGCCACACGA 3′ and 5′ CCTTCCAGAAGGTCT 3′; Syt11: 5′ CTTGTATGGCGGGGTCTTGT 3′ and 5′ ATACGCCCCAGCTTTGATGA 3′ and GAPDH: 5′ GGAGGAAACCTGCCAAGTATGA 3' and 5' TGAAGTCGCAGGAGACAACC 3'.

### 2.8. Statistics

Data are presented as mean ± s.e.m. The level of statistical significance was established at *p* < 0.05 in one-way ANOVAs with appropriate post hoc tests and two-tailed t-tests, performed with Prism 8 software (GraphPad, ***p < 0.001, **p < 0.01, *p < 0.05, #*p* < 0.0001).

## 3. Results

### 3.1. D2 autoreceptors and the dopamine transporter limit the extent of somatodendritic dopamine overflow in mouse ventral tegmental area slices

Although detectable in other rodent models such as the guinea pig [6,7,9,28], STD DA release in mouse mesencephalic slices has previously been found to be challenging to reliably detect [19,28,29]. As mouse models are convenient for genetic manipulations, the difficulty to use this model has greatly slowed progress in better understanding the mechanisms and roles of this form of DA release. We therefore first aimed to optimize detection of STD DA release in mouse VTA slices by comparing different modes of stimulation and physiological parameters that may limit its extent.

**Figure 1.**
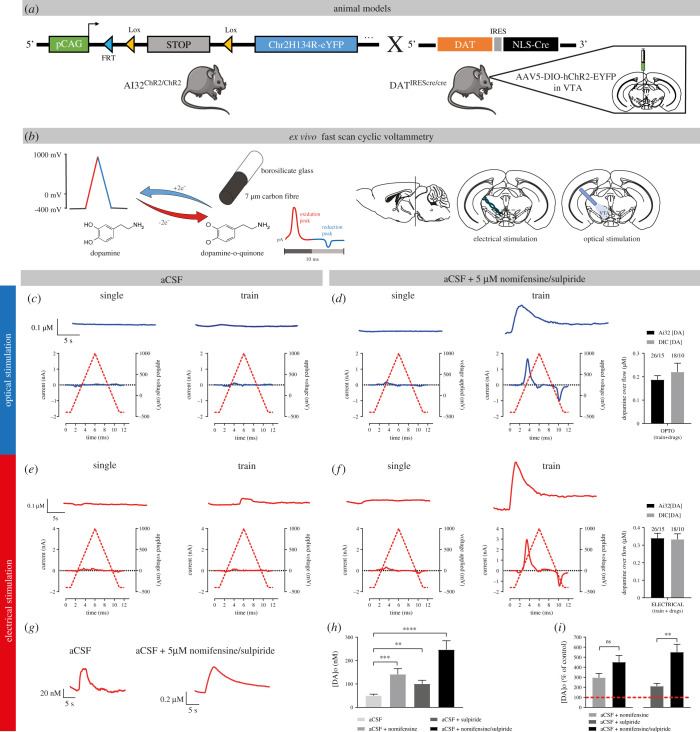
Optogenetic and electrical stimulation trigger comparable levels of STD DA release in mouse VTA slices. (*a*) Animal models used for optogenetic experiments. We either used a mouse line expressing a floxed version of light-activated channelrhodopsin (ChR2) crossed with a DA-specific Cre driver line (DAT^IREScre^) or performed stereotaxic injections of AAV5-EF1a-DIO-hChR2(H134R)-eYFP virus in the VTA of DAT^IREScre^ mice to selectively express ChR2 in DA neurons. (*b*) FSCV was used to monitor DA levels. A voltage ramp of −400 to 1000 mV versus Ag/AgCl at 300 V/s was used, with a 100 ms sampling interval. Recordings were made in coronal slices containing the VTA and DA release was triggered by either optical stimulation with a 470 nm blue light LED or with a bipolar stimulating electrode. (*c*) Representative traces (top) and voltammograms (bottom) of responses obtained in the VTA with 1 pulse (1 ms) of blue light (single pulse), in the presence of normal ACSF or a pulse train of stimulation (30 pulses of 1 ms at 10 Hz), in the presence of normal ACSF. (*d*) The same as (*c*), in the presence of ACSF + a DAT blocker (nomifensine, 5 µM) and an antagonist of D2 autoreceptors (sulpiride, 5 µM). (*e*) Representative traces (top) and voltammograms (bottom) of responses obtained in the VTA with 1 electrical pulse (1 ms, 400 µA) or a pulse train (30 electrical pulses of 1 ms at 10 Hz, 400 µA), in the presence of aCSF. (*f*) The same as (*e*), but in presence of aCSF + 5 µM nomifensine/sulpiride. (*g*–*i*) Effect of nomifensine/sulpiride on STD DA release measured by pulse-train electrical stimulation.

We first compared optogenetic and electrical stimulation of DA neurons and examined whether blocking DA D2 autoreceptors and the DAT might increase extracellular DA levels and make STD DA easier to detect (figure 1). Previous studies performed in brain slices or *in vivo* typically triggered STD DA release using extracellular electrical stimulation [34–36]. In recent years, optical stimulation using channelrhodopsin-2 (ChR2) or other opsin variants has increasingly been used to obtain more selective activation of DA neuron axons [37]. However, to this date, this approach has not been used to selectively trigger STD DA release in FSCV experiments.

We compared single-pulse optical (1 ms, 470 nm) and electrical (1 ms, 400 µA) stimulation. Recordings were performed in the VTA of DAT^IREScre^/Ai32 mice, in which ChR2 is conditionally expressed in all DA neurons, and in DAT^IREScre^ heterozygote mice injected in the VTA with a floxed hChR2-EYFP AAV construct. Neither stimulation conditions, either in normal aCSF (figure 1*c*,*e*) or in the presence of DAT (nomifensine, 5 µM) and D2 receptor blockade (sulpiride, 5 µM) **(**figure 1*d*,*f*) yielded detectable evoked elevations of extracellular DA (figure 1*c*,*d*). Previous works in guinea pig demonstrated that STD DA release is frequency dependent up to 10 Hz [9]. The use of pulse trains (30 pulses at 10 Hz) in the presence of nomifensine and sulpiride allowed reliable detection of STD DA release in VTA slices, both for electrical stimulation (average peak DA levels of 340 nM ± 28 nM in DAT^IREScre^/Ai32 mice [*n* = 15] and 333 nM ± 32 nM in DAT^IREScre^ mice infected with ChR2 AAV [*n* = 10]) and for optical stimulation (average peak DA levels at 187 nM ± 17 nM in DAT^IREScre^/Ai32 mice [*n* = 15] and 220 nM ± 37 nM in DAT^IREScre^ mice infected with ChR2 AAV [*n* = 10]) (figure 1*d*,*f*). Although peak levels of activity-dependent STD DA release in the two strains of mice tended to be higher with electrical compared to optical stimulation (compare bar graphs in figure 1*d*,*f*), the difference between the two modes of stimulation was significant only in the Ai32 mouse strain.

Reuptake through the DAT and the D2 autoreceptor are two well-known regulators of extracellular DA levels and DA release [38–40]. We next examined the effect of DAT and D2 receptor blockade individually to determine whether a combined block of reuptake and autoreceptor function was required to reliably detect STD DA release in response to electrical train stimulation (figure 1*h*,*i*). Each recording was performed after 15 min of nomifensine or sulpiride, or a combination of the two. Baseline levels of evoked DA in the absence of antagonist were small, but still reliably detected (49 nM ± 6 nM). Blockade of DAT or D2 receptors individually, caused a significant increase in the maximal amplitude of evoked STD DA release in the VTA (+296% ± 42% for nomifensine alone, *n* = 6; +210% ± 28% for sulpiride alone, *n* = 6), while a combination of the two drugs caused a cumulative increase of 500% ± 51% (*n* = 12), thus demonstrating that the two manipulations were mostly additive and that a combined blockade of both membrane proteins allowed to maximally increase the detected signal.

Because previous work evaluating STD DA release using FSCV in guinea pig brain slices reported the existence of notable stimulation-dependent attenuation [9], we also examined the stability of STD DA release in response to a series of seven consecutive stimuli with an interstimulus interval of 5 min. For these experiments, optical or electrical train stimulations were used in the presence of nomifensine and sulpiride. We found that while STD DA overflow evoked by optical stimulation showed a robust and progressive decrease in peak amplitude in response to repeated stimuli (electronic supplementary material, figure S2A), electrical stimulation failed to cause a similarly extensive rundown of STD DA, with only a modest, non-significant decrease of less than 20% detected by the last of seven stimuli (electronic supplementary material, figure S2B). Additional experiments would be required to identify the causes of this differential stimulation-dependent attenuation for the two modes of stimulation. All further experiments were performed with electrical stimulation.

### 3.2. Dopamine release in the ventral tegmental area and striatum exhibit similar calcium dependency

**Figure 2.**
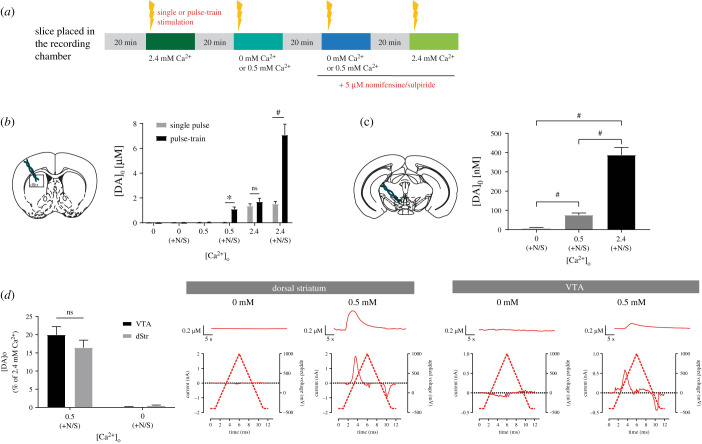
STD and axonal DA release exhibit a similar calcium dependency. (*a*) Protocol used for FSCV recordings. (*b*) Schematic representation of a striatal slice and average of [DA]o peaks obtained with single or pulse-train stimulations at 0, 0.5 and 2.4 mM of extracellular calcium in the aCSF, with or without the addition of 5 µM of nomifensine/sulpiride. (*c*) Schematic representation of a VTA slice and average [DA]o peaks obtained with pulse-train stimulations at 0, 0.5 and 2.4 mM of extracellular calcium in the aCSF containing 5 µM of nomifensine/sulpiride. (*d*) Average [DA]o peaks normalized to 2.4 mM of calcium obtained in the VTA and dorsal striatum (dStr) with pulse-train stimulation and aCSF containing nomifensine/sulpiride. Representative traces and voltammograms are shown on the right. Error bars represent ± s.e.m. The statistical analysis was carried out by a one-way ANOVA followed by a Dunnett test (ns, non-significant; ***p < 0.001; #*p* < 0.0001).

As we aimed to examine the role of Syt calcium sensors in STD DA release and in the face of conflicting previous results regarding the extent to which STD DA release depends upon extracellular calcium levels in comparison with axonal release [19,41], we next evaluated the release of DA in the VTA at 0, 0.5 and 2.4 mM of extracellular calcium (figure 2*a*). In comparison, we also examined axonal DA release in the dorsal striatum, a region that has been most closely examined in this respect. As expected, based on previous results [17,19], no release was detected in the striatum at 0 mM and 0.5 mM calcium, neither in response to single pulses or to trains (*n* = 13 slices/7 mice) (figure 2*b*).

In the VTA, STD DA release, here again triggered in the presence of nomifensine/sulpiride (5 µM), was also undetectable at 0 mM extracellular calcium (*n* = 13 slices/7 animals), but readily detectable at 0.5 mM (*n* = 16 slices/10 mice) (figure 2*c*), as previously described in the guinea pig [19,41]. Evoked STD DA release at this concentration of calcium was, however, only 19% of the signal detected at 2.4 mM calcium (76 nM ± 10 nM, compared to 388 nM ± 39 nM) (figure 2*c*,*d*), which is compatible with previous results [19]. Recordings performed in striatal slices in the presence of nomifensine and sulpiride similarly revealed detectable DA release at 0.5 mM calcium (figure 2*b*) (1.1 µM ± 0.17 µM, *n* = 13 slices/7 mice). This represents 16% of the DA signal detected at 2.4 mM calcium (7 µM ± 0.87 µM) (figure 2*b*,*d*). Therefore, under the same experimental conditions, with no influence of DA uptake and D2 autoreceptor activation, evoked STD and axonal DA release show a similar calcium dependency (*t*-test, *p* = 0.2744). All further experiments were performed at 2.4 mM calcium.

### 3.3. Dopamine neurons *in vivo* express the calcium-sensors synaptotagmin 1, 4 and 7

Because Syt1 is the main calcium sensor of axonal DA release [3,42,43], and Syt4 and Syt7 were previously suggested to be critical for STD DA release based on *in vitro* experiments [3], we next evaluated the presence and subcellular localization of these Syt isoforms in DA neurons *in vivo* in the mouse brain.

**Figure 3.**
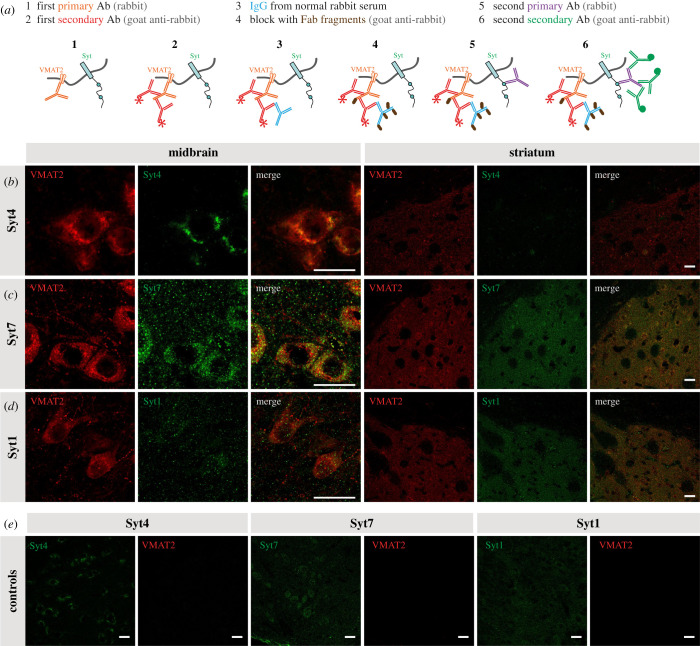
DA neurons express the calcium-sensors Syt1, Syt4 and Syt7. (*a*) Protocol used for double immunostaining for two primary antibodies from the same host (adapted from a Jackson ImmunoResearch protocol). Use of normal rabbit serum and unconjugated Fab fragments for blocking after the first secondary. (*b*–*d*) IHC of midbrain and striatal slices of adult DAT^IREScre^ heterozygote mice showing colocalization of VMAT2 and either Syt1, Syt4 or Syt7 in DAergic neurons. Scale bar = 20 μm. (*e*) Control images were obtained using the full protocol without the use of the second primary antibody (in the midbrain).

IHC was used to test the hypothesis that Syt4 and Syt7 are present within the cell body and dendrites of DA neurons in close association with compartments containing VMAT2. Due to the impossibility to obtain suitable VMAT2 and Syt antibodies produced in different species, we took advantage of a double labelling protocol allowing the use of two primary antibodies from the same species (rabbit) (figure 3*a*). The approach was validated by the observation that in control experiments in which the second primary antibody was omitted, no signal was detected for the second antigen, demonstrating that the second secondary antibody was unable to bind to the first primary antibody after the blocking step. Immunoreactivity for Syt4 showed a clear somatic localization in DA neurons, with a notable overlap with VMAT2 (figure 3*b*), with little if any signal in terminals in the striatum. Confirming the specificity of the antibody, signal was absent from Syt4 KO DA neurons (electronic supplementary material, figure S1A). Syt7 immunoreactivity was found in both the STD region of DA neurons as well as in their terminal region in the striatum (figure 3*c*). Syt7 immunoreactivity was strongly reduced in Syt7 KO tissue, although some background signal was still detectable (electronic supplementary material, figure S1B), suggesting sub-optimal specificity. Finally, Syt1 was not readily detectable in the soma and dendrites of DA neurons (although present in local VMAT2-negative axonal-like varicosities), but highly expressed in the terminals in the striatum, as expected (figure 3*d*).

### 3.4. Double knockout of Syt4 and Syt7 strongly reduces somatodendritic dopamine release

**Figure 4.**
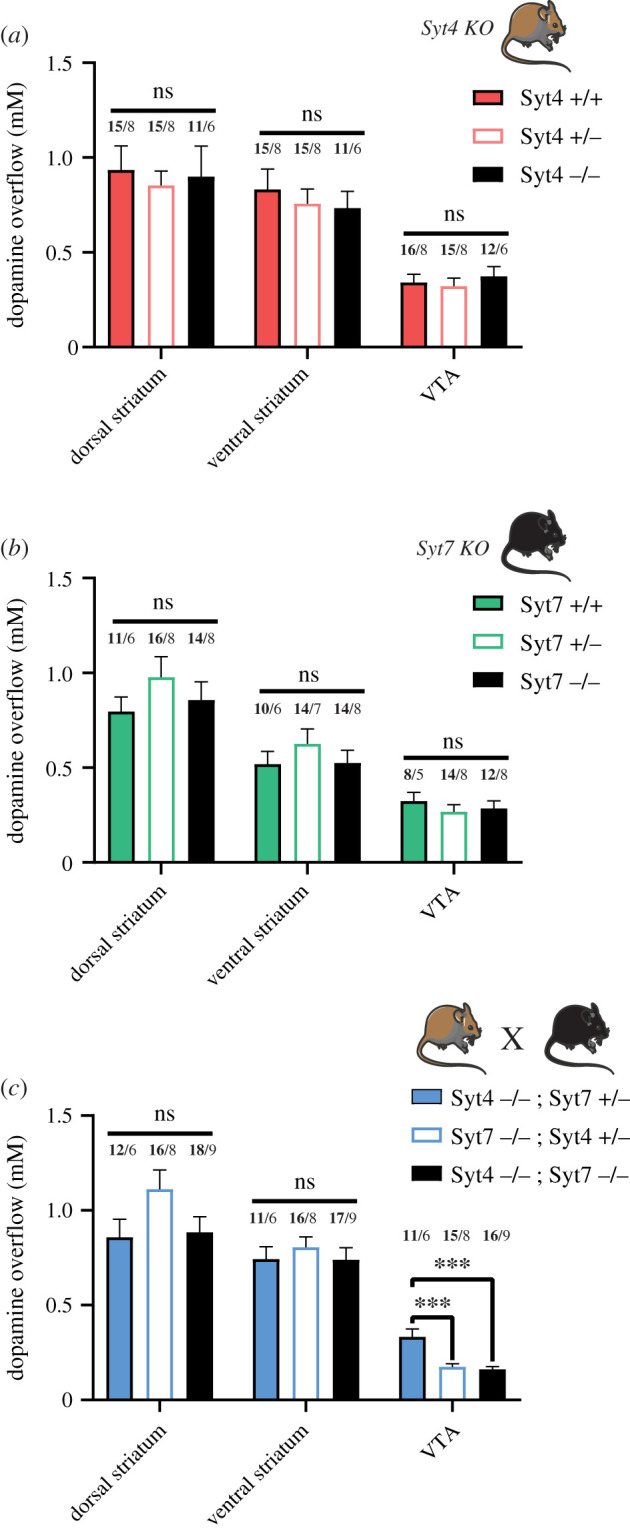
Double KO of Syt4 and Syt7 strongly reduces STD DA release. (*a*) Average [DA]o peaks (µM) obtained in the dorsal striatum, ventral striatum and VTA of Syt4 constitutive KO mice bred from heterozygous crosses. (*b*) Same for the constitutive Syt7 KO mice. (*c*) Same for double Syt4/Syt7 KO mice (Syt4^−/−^; Syt7^−/−^) and heterozygotes control animals (Syt4^−/−^; Syt7^+/−^ and Syt7^−/−^; Syt4^+/−^). Each recording from the VTA was obtained in aCSF+5 μM nomifensine/sulpiride with two recording sites per slice, two slices per animal and pulse-train stimulation (30 pulses, 10 Hz, 400 µA). Each recording from the striatum was obtained in normal aCSF from six recording sites per slice, two slices per animal, for the dorsal striatum and four for the ventral striatum (nucleus accumbens core and shell), using single-pulse stimulation (1 ms, 400 µA). Error bars represent ± s.e.m. The statistical analysis was carried out by one-way ANOVA followed by a Tukey test (ns, non-significant; ***p < 0.001; #*p* < 0.0001). Numbers above bars represent the number of slices recorded (in bold) followed by number of animals used.

Considering the expression of Syt4 and Syt7 in DA neurons and their apparent localization in the STD compartment of these neurons, we hypothesized that evoked STD DA release should be reduced in constitutive Syt4 or Syt7 KO mice. We took advantage of existing mouse models in which the Syt4 and Syt7 genes were interrupted by the insertion of a neomycin cassette in the coding sequences of the calcium-binding domains [30,31]*.* These experiments were performed using electrical train stimulation, in the presence of nomifensine and sulpiride. To obtain a thorough understanding of the individual roles of Syt4 and Syt7, wild-type, heterozygous and KO littermates were compared, and recordings were performed for each mouse in the dorsal striatum, the ventral striatum (nucleus accumbens core and shell) and the VTA. These experiments revealed that axonal DA release in the striatum and STD DA release in the VTA were not significantly reduced in Syt4 or Syt7 KO mice (figure 4*a*,*b*).

Because both isoforms are expressed in the STD domain of DA neurons, we aimed to determine if compensatory upregulation of the Syt isoforms occurred in individual KO mouse lines during development. Using qPCR and mRNA obtained from whole brain, we found that total levels of Syt4 mRNA were unchanged in Syt7 KO mice (electronic supplementary material, figure S1D). Similarly, the levels of Syt7 mRNA were unchanged in Syt4 KO mice (electronic supplementary material, figure S1E). To examine if functional compensation can explain the lack of change in STD DA release in the single KO mice, we next crossed these two mouse lines to generate a double Syt4 and Syt7 KO. As controls, Syt4^−/−^; Syt7^+/−^ and Syt7^−/−^; Syt4^+/−^ animals were also used for the FSCV recordings. Once again, no significant differences were found in the dorsal and ventral striatum (figure 4*c*). The evoked increase in extracellular DA concentration in the VTA of Syt4^−/−^; Syt7^+/−^ (0.334 µM ± 0.04 µM; *n* = 6 mice) was similar to controls from the Syt4 and Syt7 individual KO mouse lines (respectively 0.341 µM ± 0.044 µM; *n* = 8 mice and 0.32 µM ± 0.046 µM; *n* = 5 mice). However, in Syt4^−/−^; Syt7^−/−^ animals, we found a robust and significant approximately equal to 50% decrease of STD DA release (0.162 µM ± 0.014 µM; *n* = 9 mice). Interestingly, the Syt7^−/−^; Syt4^+/−^ animals also showed a systematic decrease of STD DA release of about approximately 50% (0.175 µM ± 0.016 µM; *n* = 8 mice). Together these results argue that both Syt4 and Syt7 isoforms contribute to STD DA release, with functional compensation of one isoform by the other. These data also suggest a more critical role of Syt7 compared to Syt4 because the presence of only one Syt7 allele is sufficient to support STD DA release in the absence of Syt4.

## 4. Discussion

### 4.1. Characteristics of somatodendritic dopamine release

In the present study, we performed the first characterization of optically evoked STD DA release in the mouse mesencephalon using a combination of optogenetics and FSCV and compared its characteristics to release evoked by electrical stimulation. As previously reported by others, we found that the absolute levels of evoked DA overflow detected in this region were low compared to levels detected in the terminal region in the striatum. Furthermore, we found that a robust STD DA release signal could only be detected using pulse-train stimulation (figure 1). Blocking DA reuptake and D2 autoreceptor function using nomifensine and sulpiride caused a fivefold increase in peak signal amplitude, thus making detection of this signal straightforward and reproducible.

Using a repeated stimulation protocol, we found that repeated optical stimulation with a 5 min interval produces a strong rundown of STD DA release, whereas no such attenuation was seen with electrical stimulation (electronic supplementary material, figure S2). This finding is compatible with previous results reporting a similar rundown of optically evoked axonal DA release in the striatum [37]. Although a partial desensitization of ChR2 upon repeated stimulations could occur [44,45], a more likely explanation of the different rate of decrement obtained with optical and electrical stimulation is that in response to electrical stimulation, a decrement is not observed because this form of stimulation recruits neuromodulatory mechanisms that result from activation of afferent terminals releasing 5-HT, NE, acetylcholine, glutamate, GABA or neuropeptides onto DA neurons [37,46]. Further experiments will be required to test this hypothesis.

Our experiments comparing the impact of changes in extracellular calcium levels of STD and axonal DA release argue for the existence of a similar calcium dependency for both forms of release (figure 2). These findings are compatible with previous results obtained in mice, which were performed using patch-clamp recordings and the measurement of STD D2 receptor-mediated membrane currents [19]. It is possible that a different conclusion was reached in guinea pig brain slices because some aspect of the STD DA release mechanism is different in that species [17], as previously suggested [28]. Another possibility is that a component of axonal DA release is also included in the signal detected in the VTA. This possibility has been raised previously [41], but the available anatomical data actually suggests that DA-containing axonal varicosities are scarce in the VTA [47,48], except in the context of compensatory axonal sprouting associated with partial lesions [49]. It would nonetheless be useful to revisit this question in the future with additional anatomical and physiological work to provide more quantitative data.

### 4.2. Contribution of Syt4 and Syt7 to somatodendritic dopamine release

Finally, we examined the contribution of the Syt isoforms Syt4 and Syt7 to STD DA release. Both Syt4 and Syt7 have been linked to vesicular fusion. A role for Syt7 in asynchronous release was previously demonstrated [50]. This isoform has also been shown to synergize with Syt1 [51]. The function of Syt4 is more controversial because it has been proposed to act either as a negative regulator of vesicular exocytosis [52–54] or to promote some forms of neurotransmitter release [3,55,56], depending on the system investigated. In posterior pituitary nerve terminals, such positive and negative roles were reported to depend on calcium concentrations, suggesting that the function of Syt4 can be modulated according to physiological conditions [57].

In the dopaminergic system, acute downregulation of either isoform has previously been shown *in vitro* to severely reduce STD DA release, with no similar effect of Syt1 downregulation [3]. Although our present immunostaining results provide further support for the presence of these proteins in the STD compartment of DA neurons (figure 3), we failed to detect any significant decrease in evoked STD DA release in VTA slices prepared from individual constitutive Syt4 or Syt7 KO mice (figure 4). It is possible that, contrary to acute downregulation with siRNAs, constitutive gene deletion may lead to homeostatic compensation leading to elevated levels of Syt4 in Syt7 KO mice and vice versa. It is also possible that *in vitro* models lack homeostatic compensatory mechanisms that are recruited *in vivo*. Another possibility is that Syt4 and Syt7 play similar roles in supporting STD DA release and that one can compensate for the absence of the other in the context of constitutive gene deletion. The robust decrease in activity-dependent STD DA release in Syt4/Syt7 double KO mice supports this interpretation. In Syt4^−/−^; Syt7^−/−^ mice or Syt7^−/−^; Syt4^+/−^ mice, we observed a twofold decrease of STD DA release. This decrease was surprisingly not found in Syt4^−/−^; Syt7^+/−^ mice, strongly suggesting that Syt7 plays a particularly important role and that a single allele of Syt7 is sufficient to sustain STD DA release in the absence of Syt4. This observation of a particularly important role of Syt7 in STD DA release could be linked to differential age-dependent expression of Syt7 and Syt4 in DA neurons. Indeed, the level of expression of Syt7 was previously demonstrated to increase as a function of postnatal age in mice [3]. Conversely, the expression of Syt4 in the brain has been shown to be maximal during the first postnatal week and to slowly decline thereafter [58,59]. Here, we conducted all experiments in adult animals, at a developmental stage where Syt7 may play a more important role in regulating STD DA release compared to Syt4. Further experiments comparing STD DA release in Syt4 and Syt7 double KO mice at different ages would thus be of interest in the future.

Considering that in the absence of both Syt4 and Syt7, approximately half of total evoked STD DA release remain, we also hypothesize that other calcium sensors or other forms of release are also involved. The main Syt isoform Syt1 might be of interest, as it was recently demonstrated as the main calcium sensor for fast striatal DA release *in vivo* [42], validating previous work showing an important role of Syt1 in axonal DA release *in vitro* [3]. Interestingly, evoked STD DA release measured by detecting D2-IPSCs was recently reported to be abolished in mice with conditional deletion of the active zone protein RIM, while spontaneous release remained intact [21]. However, the subcellular localization of RIM in the STD compartment of DA neurons is currently undetermined. Here, we have not found strong evidence for localization of Syt1 in the STD domain of DA neurons, but further examination of this possibility with higher resolution techniques would be relevant.

Our results bring to light an important role of Syt7 in both the dendritic and axonal compartment of DA neurons. Together with our observation that the two forms of DA secretion exhibit the same calcium dependency, our findings raise the interest of further evaluating a possible contribution of an axonal compartment to DA release within the mesencephalon, in line with previous speculations in the guinea pig model [60]*.* Further studies of STD and axonal DA release in mouse models defective for combinations of Syt1, Syt4 and Syt7 would seem warranted. Together our work provides a new perspective on the mechanism of STD DA release and raises new opportunities to disentangle the respective roles of axonal and STD DA release in DA-dependent physiological mechanisms and behaviours.

## Data Availability

Primary data analysis files from this manuscript are available from the following doi:10.6084/m9.figshare.19083575. Data supporting this article have also been uploaded as part of the electronic supplementary material.
